# Development of a Solar-Powered IoT-Based Instrument for Automatic Measurement of Water Clarity

**DOI:** 10.3390/s20072051

**Published:** 2020-04-06

**Authors:** Tuan Ngoc Pham, Anh Pham Huy Ho, Tuong Van Nguyen, Ha Minh Nguyen, Nhu Huynh Truong, Nguyen Duc Huynh, Tung Huy Nguyen, Le The Dung

**Affiliations:** 1 National Key Laboratory of Digital Control and System Engineering, Ho Chi Minh University of Technology, VNU-HCM, Ho Chi Minh City 700000, Vietnam; pntuan@hcmut.edu.vn; 2 Faculty of Mechanical Engineering, Ho Chi Minh University of Technology, VNU-HCM, Ho Chi Minh City 700000, Vietnam; 1652431@hcmut.edu.vn (N.D.H.); 1652683@hcmut.edu.vn (T.H.N.); 3 Faculty of Electrical and Electronics Engineering, Ho Chi Minh University of Technology, VNU-HCM, Ho Chi Minh City 700000, Vietnam; hphanh@hcmut.edu.vn; 4 Faculty of Mechanical Engineering, Nha Trang University, Khanh Hoa Province 650000, Vietnam; tuongnv@ntu.edu.vn; 5 Saigon Center for Development of Industrial Technology and Machinery, Ho Chi Minh City 700000, Vietnam; ha.nguyen@cenintec.com; 6 Faculty of Electrical Engineering, Ba Ria-Vung Tau College of Technology, Ba Ria - Vung Tau Province 78000, Vietnam; nhuth@bctech.edu.vn; 7 Division of Computational Physics, Institute for Computational Science, Ton Duc Thang University, Ho Chi Minh City 700000, Vietnam; 8 Faculty of Electrical and Electronics Engineering, Ton Duc Thang University, Ho Chi Minh City 700000, Vietnam

**Keywords:** water clarity, automatic measurement, light, LED, Internet of Things, Secchi disk

## Abstract

Water clarity is the most common indicator of water quality. The purpose of the study was to develop an instrument which can automatically measure water clarity in place of manual measurement by Secchi disk. The instrument is suspended by buoys at the water surface and uses solar energy to measure the light intensity of LED bulbs after passing through a water column; the result is then converted to Secchi depth by using a regression function. Measurement data are stored in a cloud server so that mobile users can access via an Internet connection. Three experiments were conducted to examine the instrument performance: (i) to ensure light intensity of the LED bulbs is strong enough to pass through the water column; (ii) to determine the regression relationship between the measured light intensity of the instrument and Secchi depth; and (iii) to evaluate the coefficient of variation (CV) of the measured water clarity when using our instrument and a conventional Secchi disk. Experiment results show that the measured values of light intensity are stable with the average CV = 5.25%. Moreover, although there are slight differences between the Secchi depth measured by our instrument and those measured by Secchi disk, the measurements by our instrument can efficiently replace the measurements by conventional Secchi disk, which can be affected by weather conditions as well as by human subjectivity.

## 1. Introduction

Nowadays, water pollution and concerns about aquatic ecosystem and human health have become global issues. To remediate or prevent pollution, it is necessary to monitor water quality. Water clarity is the most common indicator of water quality [1]. It is a physical characteristic defined by how clear or transparent water is and how much light penetrates through water. The further light can reach, the higher the water clarity is, the deeper the photic zone is, and the greater the capacity to support the growth of aquatic plants and animals becomes [2,3]. Water clarity can also be called as “water transparency” [4], “Secchi depth” [5], “Secchi disk depth” [6], “Secchi disk transparency”, or “Secchi transparency”. The purpose of the study was to develop an instrument which can measure water clarity automatically in place of manual measurement by Secchi disk.

There are several methods to measure water clarity. The simplest and most popular method is using Secchi disk [7,8]. The Secchi disk, created in 1865 by Angelo Secchi, is a plain white, circular disk 30 cm in diameter used to measure water clarity in bodies of water. The disk is attached to a rope and lowered slowly down through the water column. The rope is usually marked in 0.5- or 1-m increments, which are counted by the observer lowering the disk into the water and keeping an eye on the disk until it disappears. The length of the submerged rope at which the disk is no longer visible is taken as the criterion of water clarity. This criterion is known as Secchi depth (ZSD) and is the unit of water clarity. A high ZSD corresponds with high water clarity, i.e., visibility through the water column. In the majority of cases, the observer records the depth when the disk disappears [6,9,10,11]. In other cases, the observer records the average depth when the disk disappears and reappears [12,13,14]. As for dimension, the Secchi disk is standardized at 30 cm diameter [9,15,16,17,18]. Some different dimensions are used such as 10 [18], 20 [19,20,21,22], 25 [23,24], 40, and 45 cm [23,24]. The deeper the disk is lowered, the bigger the disk needs to be [25,26]. As for color, the Secchi disk may be painted with all white [26,27,28,29] or with black and white quadrants [19,20,30,31]. The all-white disk is used for marine measurement, and the black and white is suitable for freshwater measurement [32]. Another method is using a horizontal black disk with a viewer box to measure the clarity in shallow water where the Secchi disk cannot be used [22,33,34]. The black disk is attached to the pole and placed in front of the viewer box. The observer uses the viewer box to observe the black disk. Then, the black disk is steadily moved farther horizontally from the viewer box by an assistant until the observer can no longer see the disk. After that, the distance between the black disk and the viewer box is recorded and denoted as YBD (unit of water clarity measured by the black disk). The size of the black disk depends on the water clarity. The underwater viewer box is made of 3 mm thick, optically clear sheet polycarbonate painted matte black on the inside except for end and side windows. The side window is used for right-angle viewing (horizontal direction in the water) with a small mirror fitted at 45${}^{\circ }$ [33]. Moreover, clarity tube can be used to measure water clarity [34,35]. The working principle of this tube is simple. First, the tube is fully filled with the water sample. Next, the first observer looks through the top of the tube while the second observer lets the water out of the tube through the valve at the bottom. As soon as the first observer can see the black and white disk at the bottom of the tube, the second observer closes the valve. Then, they record the height of the remaining water in the tube as water clarity.

Besides using the above conventional instruments, several modern instruments can be used to measure water clarity. Particularly, a nephelometer measures the light intensity that passes through the water sample [22,36], while a turbidimeter measures the scattered light intensity in the water sample [37,38,39]. Photosynthetically active radiation (PAR) measured by an underwater light sensor is a property that can also be used to determine water clarity. The underwater light sensor can precisely quantify the PAR available at a particular depth in the water column. Measurements can be converted to light extinction coefficient, *k*. Clear water has a low *k*, while turbid water has a high *k* [3,40,41]. Using satellite TM (thermal mapping) images is a modern technique to observe water clarity. This method uses algorithms which evaluate the brightness value of red and blue band taken from the images to predict the ZSD [42,43,44]. Long Arm Marine Spectrophotometer (LAMS) is a device that measures water clarity by light transmission in deep water [45]. Lidar systems use signal decay rate to estimate water optical quality [46]. In [47], the design and development of a turbidity system for monitoring drinking water in household is presented. Its operation is based on the relationship between the nephelometric turbidity units (NTU) and the output voltage of light sensor. The developed prototype is battery powered and appropriate for turbidity measurements in the range of 0–100 NTU with precision of 0.2 NTU. A methodology for the design, development, and technical validation of a low-cost, open-source (OS) water testing platform is provided in [48]. A case study is presented where the platform is battery powered to provide both the colorimetry for biochemical oxygen demand/chemical oxygen demand and nephelometry to measure turbidity using method ISO 7027.

After reviewing the methods mentioned above in the literature, we observe that, for the conventional methods, the measurement is greatly affected by observers’ subjectivity, weather conditions, and, most of all, it cannot be done continuously to get real-time data. For modern methods, the first barrier is that those instruments are expensive. They also require many skilled people to operate, and some methods are not suitable for small scale (aquaculture ponds, small lakes, etc.) measurement. Meanwhile, although the Internet of Things (IoT) system is available in water quality monitoring, water clarity sensor is currently unavailable. Motivated by the limitations of these methods, the main aim of this study was to develop a low-cost, autonomous, IoT-based instrument which uses solar energy to support the automatically measuring the water clarity in place of manual measurement by Secchi disk. The experiment results show that the instrument is convenient and can replace the Secchi disk in the water clarity measurement. Moreover, the measurement data are collected by the sensor and processed directly by the controller, and then are sent and stored in the cloud for better management decisions.

The rest of this paper is organized as follows. The main features, structure, and working principle of our proposed instrument are presented in Section 2. Experiments used to evaluate the performance of this instrument are described in Section 3. Experimental results and discussion are given in Section 4. Finally, conclusions are drawn in Section 5.

## 2. Introduction to Our Proposed Instrument

### 2.1. Main Features

**Figure 1:**
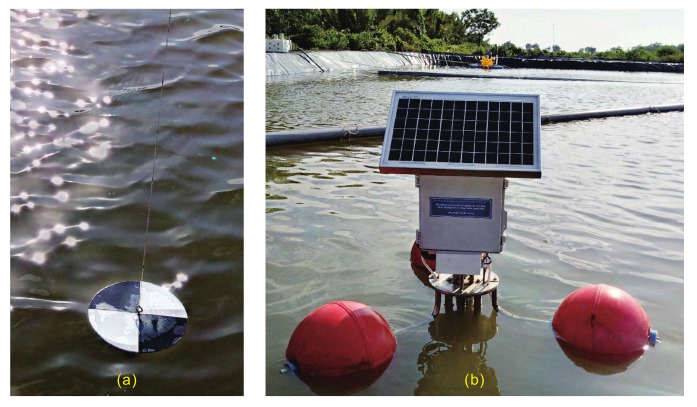
Measurement in field condition: (**a**) manual measurement by using Secchi disk; and (**b**) automatic measurement by using our proposed instrument.

Figure 1 demonstrates the measurement of water clarity by using a Secchi disk and our proposed instrument. Our proposed instrument is intentionally designed to evaluate the water clarity of aquaculture man-made ponds and lakes based on Secchi depth (ZSD). Thus, it saves measurement time and cost but still provides an accurate assessment of water clarity. The range of ZSD that our proposed instrument can measure is between 20 and 70 cm. Moreover, the instrument uses solar energy and can work autonomously, helping to reduce supervision and maintenance efforts. It can be connected with other devices in IoT applications so that users can access measurement data via an Internet connection. In the following parts, we present in detail the structure and operations of this instrument.

### 2.2. Structure

**Figure 2:**
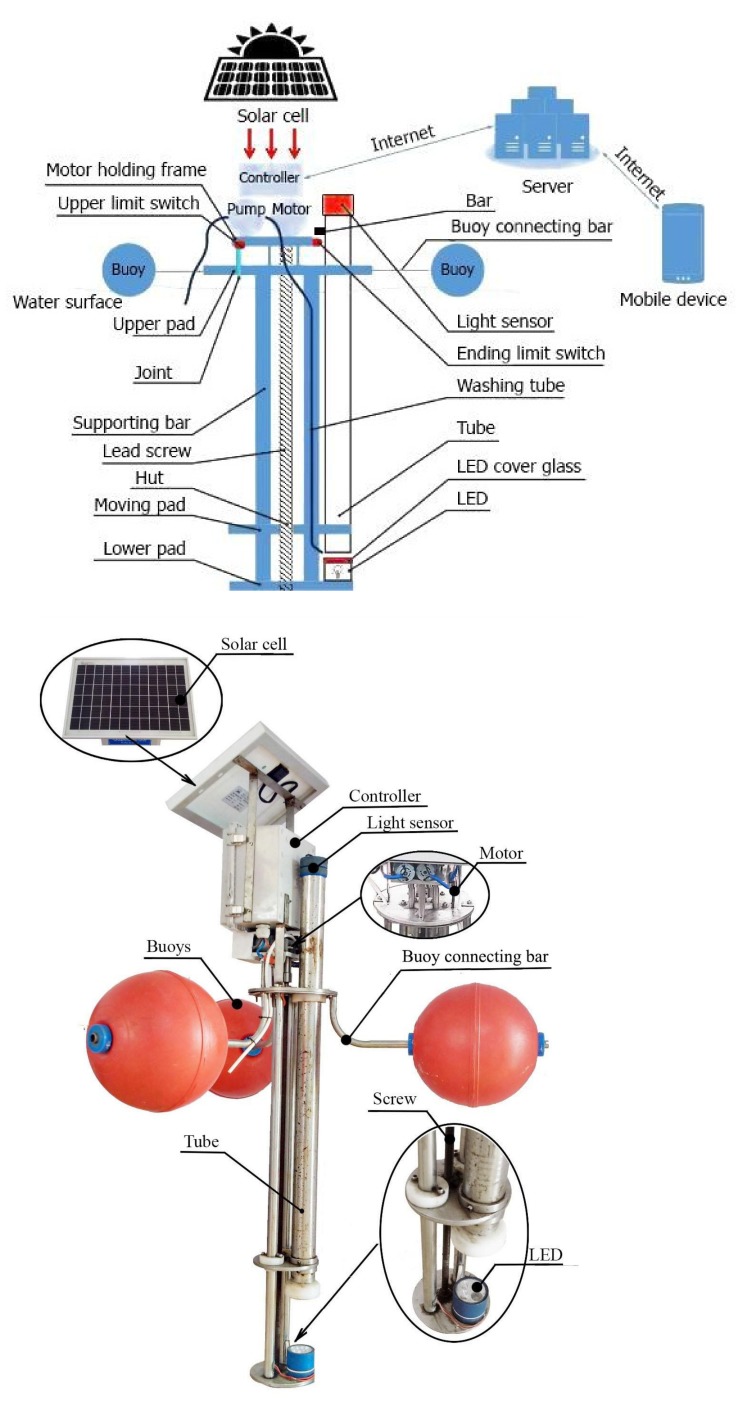
Design of our proposed instrument and its real appearance.

The design of our proposed instrument and its real appearance are presented in Figure 2. Particularly, the instrument is comprised of four assemblies as described below.

#### 2.2.1. Frame and Buoy Assembly

Frame and buoy assembly are used to fix all components and keep the instrument floating on water surface. For operating in different types of water, the frame is manufactured by 304 stainless steel. It consists of several main parts such as an upper pad, a lower pad, three buoys, a supporting bar, a motor holding frame, and three buoy connecting bars. The upper pad holds a controller plastic box, a motor, and the solar cell. The lower pad is welded to the upper pad by two supporting bars. The motor used for moving the tube up-and-down and for running the washing pump is installed on a motor holding frame. Three plastic spherical buoys are connected with three connecting bars to keep the instrument floating on the water surface for efficiently collecting water samples. This buoy assembly is designed so that it can support the instrument in working independently. However, when necessary, our instrument can be attached to existing buoys on lakes without affecting the normal operations of the instrument.

#### 2.2.2. Measuring Assembly

Measuring assembly includes a lead screw, a tube, a moving pad, a light sensor, a motor, a pump, a limit switch, and LED bulbs installed in a LED box with a glass cover on top. LED bulbs (12 V, 60 W) are used as the light source of the instrument. The moving pad holding a 90-cm-long tube with a light sensor on the top can move up and down thanks to the lead screw driven by a motor to collect water samples for measuring water clarity. Upper and lower limit switches are used to limit the moving of the tube, ensuring that the tube only moves in a desired range. A 24 VDC, 6800 rpm motor is attached to a 60:1 reduction ratio gearbox connected to the lead screw drive via a shaft coupling. The screw drive made of 304 stainless steel transforms rotational motion (113 rpm) into linear motion (moving up-and-down).

Light sensor TSL2560 (Texas Advanced Optoelectronic Solutions, TX) is placed on top of the tube to measure the light intensity emitted by the LED box at the bottom of the tube. The TSL2560 can detect a light intensity range of 0.1–40,000 lux. Moreover, the tube is made of stainless steel with 50 mm diameter and 1 mm wall thickness. The inside cylindrical surface of the tube is shiny for light reflection. At the end of the tube, a plastic ring is installed to prevent light from coming out from the tube during measurement. For cleaning the tube, a 12 V mini pump is used to generate a water flow of 4 L/min at 0.2 MPa.

#### 2.2.3. Power Assembly

The instrument mainly works in ponds and lakes far from electrical power supply to protect local aquatic animals from harm. Therefore, we use a RS–P318–10W poly-crystalline PV solar power panel to supply power for the instrument. This solar panel has high-efficiency cells with long-term output stability. It is also made of low iron high transmittance tempered glass to provide better stiffness and impact resistance. The solar panel is mounted on the top of the instrument to get as much sunlight as possible. Harvested solar energy is stored in a 12 V rechargeable lithium ion battery- 12 V, 7 Ah when the instrument is idling for later utilization.

#### 2.2.4. Control Assembly

**Figure 3:**
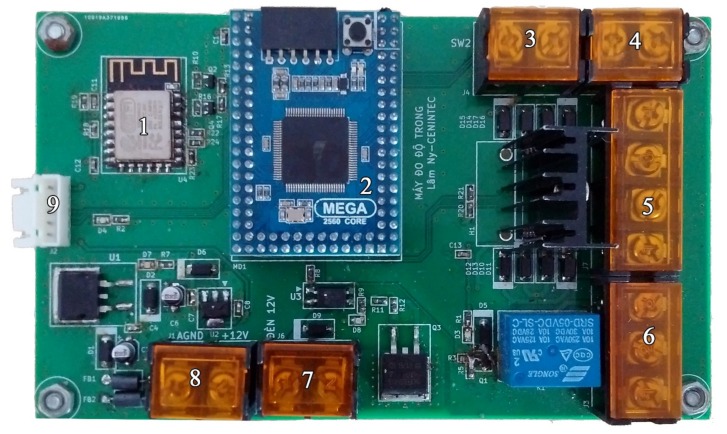
The hardware of the controller: (1) serial WiFi module ESP8266; (2) Arduino Mega2560-CORE; (3 and 4) switch ports; (5 and 6) connecting ports to motors; (7 and 8) 12 VDC power supply ports; and (9) TSL2560 light sensor jack connector.

**Hardware:** The controller is made based on Arduino Mega 2560-CORE (Inhaos, Dongguan, China). Arduino Mega2560-CORE is a small, complete, and breadboard-friendly board based on the ATMega2560 (Inhaos, Dongguan, China, 2019). This controller consists of nine components, as shown in Figure 3. Its design is based on the Arduino Mega2560, meaning that it can be used as an Arduino Mega2560 development board. The USB connection has been replaced by a UART port. The Arduino Mega2560-CORE is connected to a customized breadboard having a serial WiFi module ESP2886, two switch ports, two ports to connect to the 12 VDC motor, supply ports, and connecting jack to the light sensor. The serial WiFi module ESP2886 is integrated into the breadboard for connecting with the WiFi signal from a router to transmit all measurement data to a cloud server.

TP-link TL-MR6400 router is used for its robust wireless broadband, providing a closed connection between the instrument and cloud service. This router has a built-in modem and a 4G/LTE sim card (to be inserted into the modem) to connect to the Internet wirelessly through fast-moving mobile broadband. Thanks to the wide range and speed of 300 Mbps WiFi, the connection between server and machine is stable for the whole procedure. In addition to the router, the serial WiFi module ESP8266 is used because of its compatibility with Arduino. It is a low-cost WiFi chip with full TCP/IP capability. This board has an MCU (Micro Controller Unit) integrated, which gives the possibility to control I/O digital pins via simple and almost pseudo-code similar to a programming language. When measurements are completed, the measured data are transferred directly from the instrument to a server via WiFi connection and displayed on a user interface so that users can easily monitor water clarity anywhere.

**Figure 4:**
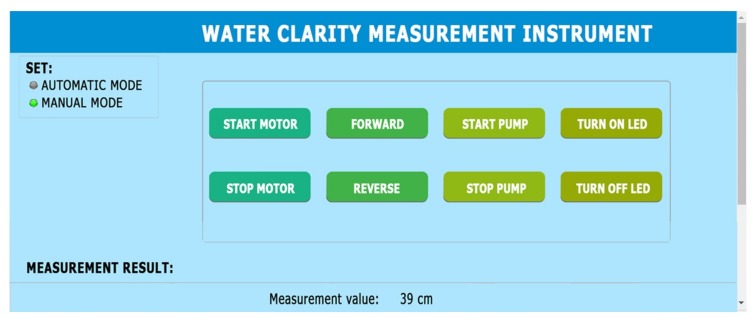
User interface for controlling the instrument from a computer via a WiFi modem.

**User interface:** The layout of user interface for controlling the instrument from a computer via a WiFi modem is shown in Figure 4. It is designed to be friendly for users to do various operations, such as controlling the device in manual or automatic mode, turning ON/OFF the motor, adjusting the rotation direction of the motor to move the measuring tube up-and-down, turning ON/OFF the washing pump and the LED bulbs, and displaying the measurement results.

**Figure 5:**
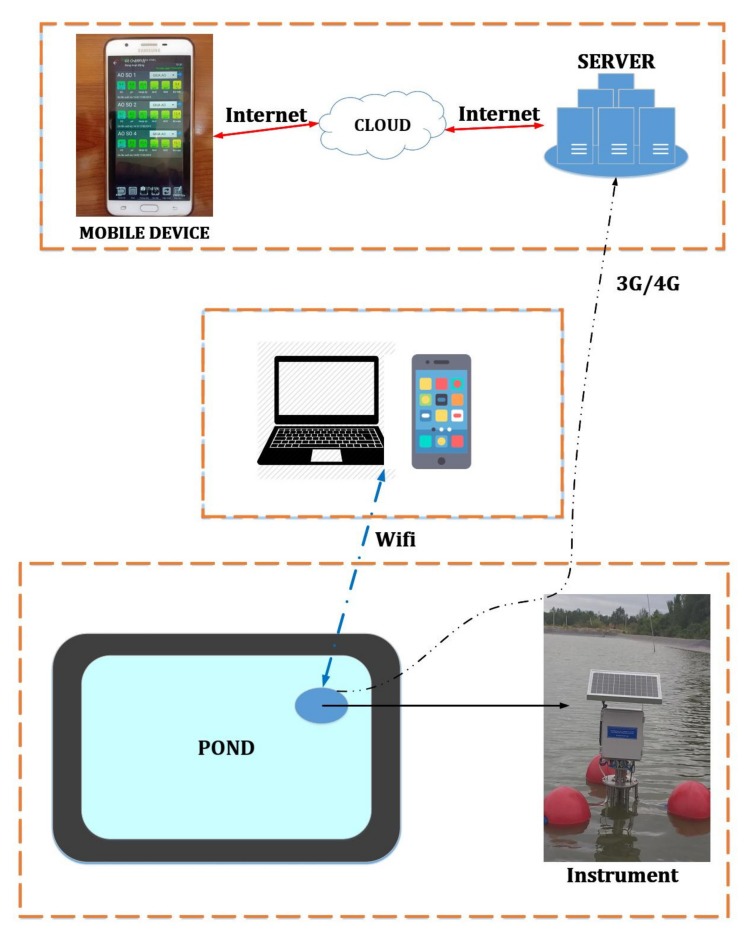
IoT application of our proposed instrument.

**IoT application:** The illustration of how our proposed instrument is connected with other devices in IoT applications is given in Figure 5. Users can use computers or mobile phones to control the instrument via a WiFi modem. Measurement data are transferred to a computer via WiFi modem or to a server via 3G/4G/LTE modem. These data are also stored in a cloud server so that users with mobile devices can access via an Internet connection.

### 2.3. Working Principle

**Figure 6:**
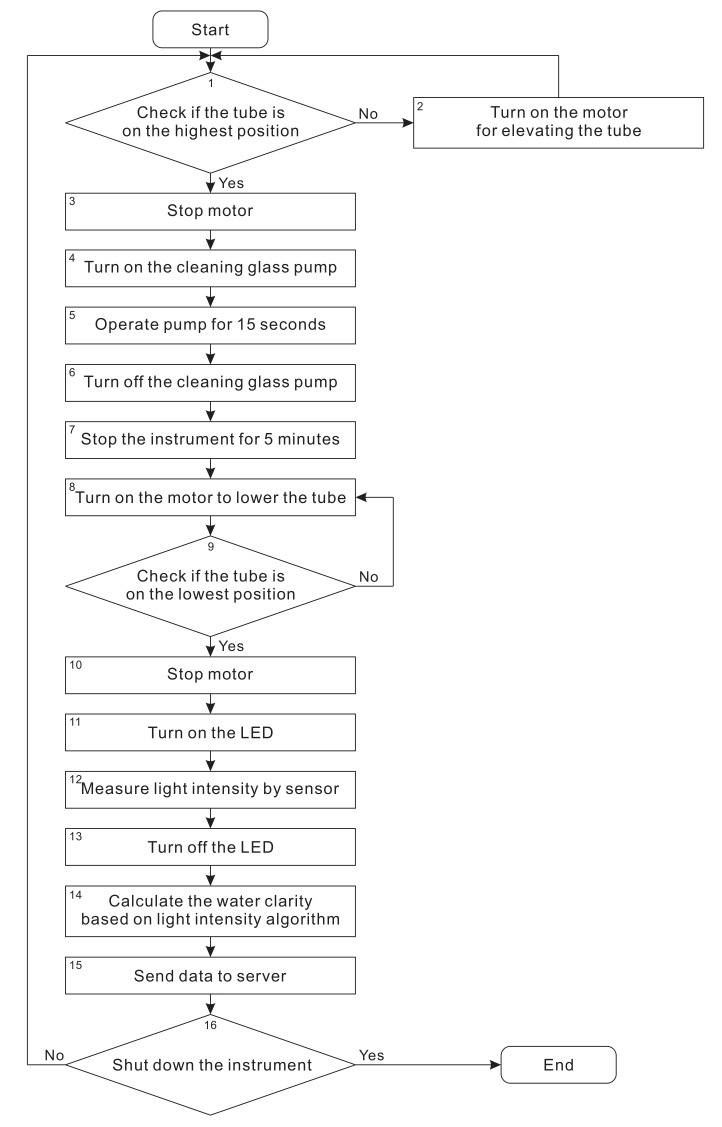
Working principle of the instrument.

The working principle of our proposed instrument is described in Figure 6. When the instrument starts, the tube moves up to the highest position. The next steps are described as follows.

**Step 1:**  Check if the tube is at the highest position. If yes, move to Step 3. Otherwise, move to Step 2.
**Step 2:**  The upper-limit switch remains “open” status when the upper pad does not touch the upper-limit switch’s joint so that the tube keeps elevating until the upper pad touches the joint.
**Step 3: **  When the upper pad touches the upper limit switch’s joint, the controller receives “close” status to turn off the motor.
**Step 4: **  After turning off the motor, the controller operates the mini pump to clean the cover glass of LED box. This process helps to avoid dirty substances on the glass surface which reduces the efficiency fo light transmission.
**Step 5: **  The pump operates for a duration of 15 s. This is the time for the pump to clean the cover glass of LED box.
**Step 6: **  After 15 s, the pump is turned off.
**Step 7: **  As soon as the pump is switched off, the controller stops the instrument for a duration of 5 min. During this period, the current water sample is replaced by a new one. Then, the next measurement result corresponding with the new water sample is recorded.
**Step 8: **  After 5 min, the controller turns on the motor to elevate the tube below water surface to create an isolated measuring environment so that external factors such as weather conditions cannot affect the accurateness of measurement results.
**Step 9:**  Check if the tube is at the ending position or not? If yes, move to Step 10. Otherwise, move back to Step 8.
**Step 10:**  For moving down, the lower-limit switch remains “open” status when the upper pad does not touch the upper-limit switch’s join so that the tube keeps elevating until the upper pad touches the joint. The “open” status is switched to the “close” status. Then, the controller recognizes this event and turns off the motor.
**Step 11:**  Right after turning off the motor, the controller turns on the LEDs to begin the measuring process for the new water sample filled in the tube.
**Step 12:**  As long as the LED bulbs are on, the light sensor will capture light intensity passing through the water column.
**Step 13:**  After the controller receives all the results captured by the light sensor, it turns off the LED bulbs and analyzes these results.
**Step 14:**  The controller starts to calculate water clarity by using an algorithm that converts light intensity into ZSD.
**Step 15:**  The controller sends measurement results to server over an Internet connection. Users can monitor these results on computers or mobile phones.
**Step 16: **  The controller repeats the above measuring process according to a fixed routine and shuts down when it receives orders from users.

## 3. Performance Evaluation of Our Proposed Instrument

This section describes several experiments that we used to evaluate the performance of our proposed instrument.

### 3.1. Experiment of Light Intensity Measurement

**Figure 7:**
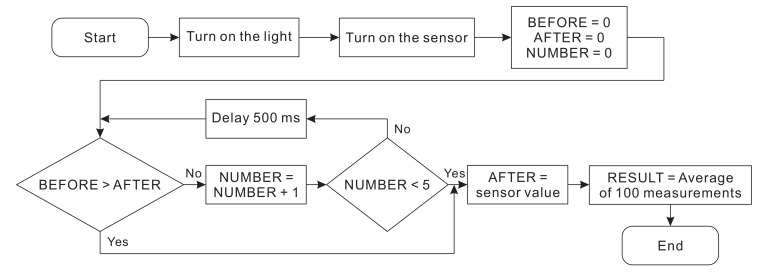
Algorithm of data acquisition from the light sensor.

Experiment of light intensity measurement was implemented to evaluate the stability of the instrument via coefficient of variation (CV). It is a cross-check experiment (i.e., the experiment whose result is the average value of many measurements taken in different locations by different measurement methods and are compared with each other), carried out at several ponds of a shrimp farm in Can Gio District, Ho Chi Minh City, Vietnam. Firstly, the measurement process was accomplished with different light intensity by placing 2, 4, 8, and 16 transparent plastic sheets on the cover glass of LED box. The algorithm for getting light intensity is shown in Figure 7. The light intensity was taken from the light sensor every 500 ms. The current result (denoted as AFTER) was compared with the result from previous cycle (denoted as BEFORE). The moment when AFTER was higher than BEFORE indicated that the measurement result started to stabilize. This moment should be caught five times for certainty. Next, an average of 100 measuring values was taken as the final result light intensity. We developed software to realize the above algorithm and carried out many measurements to get the accurate values of light intensity. Particularly, these measurements were repeated 30 times for each value of light intensity (different light intensities were created by changing the number of transparent plastic sheets covering the LED).

### 3.2. Experiment for Generating Regression Function

This experiment aimed to determine the regression relationship between light intensity and ZSD. It is also a cross-check experiment, performed at Can Gio District, Ho Chi Minh City. Firstly, ZSD was measured by ten observers with naked eyes using a 20 cm diameter black and white quadrant disk at 11:00 in sunny weather. Each observer recorded the average of the depth at which the disk disappeared and reappeared. Secondly, the mean value of ten ZSDs measured by all observers was taken. The process was repeated six times, with an increase in water clarity at six different locations. Finally, the instrument was used to measure the light intensity at the same position as the observers did. Ten data values were collected to get the mean value of light intensity. All the data of manual measurements and our instrument based measurement were noted. From the measured data, a regression function showing the relationship between the mean ZSD and the light intensity was generated by using the trendline tool in Microsoft Excel. This regression function was then used in a user interface developed in C++ to display the ZSD converted from the measured light intensity.

### 3.3. Experiment with the Instrument in Different Locations

The fast development of aquaculture in recent years leads to an increase in environmental issues. Therefore, monitoring water quality parameters, particularly water clarity, is essential for environmentally sustainable development. Vietnam is known as a country which exports vast amounts of seafood, especially shrimp. Thus, we selected some shrimp farms at Can Gio District, Ho Chi Minh City for measuring water clarity. After testing all functions, the instrument was taken to six different shrimp farms to measure their water clarity. The installation of the manual measurement with Secchi disk and the measurement with our proposed instrument is illustrated in Figure 1a,b, respectively. Six sites were chosen with different usage purposes such as a reservoir, a shrimp pond, and a wastewater pond. Each site was measured ten times both by ten different observers and by our instrument to determine the differences between these two measuring methods.

## 4. Results and Discussion

This section provides the results and discussions of several experiments presented previously. All values listed below are means ± standard deviations. Additionally, the following criteria are used in the comparisons between measurements by using our instrument and manual measurements by using Secchi disk.

- *Absolute difference* is the difference between the ZSD measured by using our instrument and the ZSD measured manually by using Secchi disk, i.e.,
  (1) $$\Delta =\left|\mathrm{ZS}D_{\mathrm{instrument}}-\mathrm{ZS}D_{\mathrm{manual}}\right|.$$
- *Percentage difference* is the ratio of Δ over $\mathrm{ZS}D_{\mathrm{instrument}}$, i.e.,
  (2) $$\Delta_{p}=\frac{\Delta }{\mathrm{ZS}D_{\mathrm{instrument}}}\times 100.$$
- *Average percentage difference* is the mean value of “*percentage difference*”.

### 4.1. Results of Light Intensity Measurement

**Figure 8:**
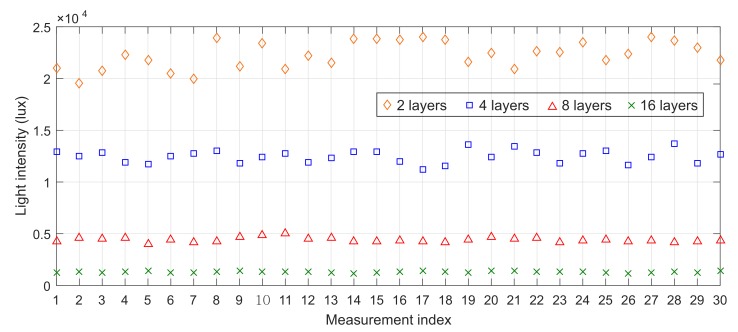
Light intensity after passing through 2, 4, 8, and 16 transparent plastic sheets in 30 measures.

The measurement results are plotted in Figure 8. We can observe the behavior of light intensity as follows: 22,277 ± 1314 lux (CV = 5.8%) in the case of using two plastic sheets, 12,423 ± 584 lux (CV = 4.6%) in the case of using four plastic sheets, 4413 ± 223 lux (CV = 4.9%) in the case of using eight plastic sheets, and 1316 ± 76 lux (CV = 5.7%) in case using sixteen plastic sheets. To sum up, the measured value of light intensity is stable with the average CV = 5.25%. In other words, the instrument works consistently with different light intensities.

### 4.2. Results of Measurement of Secchi Depth and of Light Intensity by the Instrument to Generate the Regression Function

The measured data of Secchi depth (ZSD) and light intensity (LI) are shown in Table 1. We can see tha the ZSDs from ten different observers are 17 ± 1.8, 28.55 ± 2.49, 38.85 ± 1.3, 49.4 ± 2.06, 59.3 ± 2.59, and 69.85 ± 2.3 with the CVs range of 2.83% – 10.59%. The mean light intensity measured by the instrument are 15.4 ± 0.92, 95.8 ± 1.9, 183 ± 5.18, 677.3 ± 20.7, 1,656.2 ± 44.0, and 3,855.4 ± 204 with the CVs range of 2% – 5.95%.

**Figure 9:**
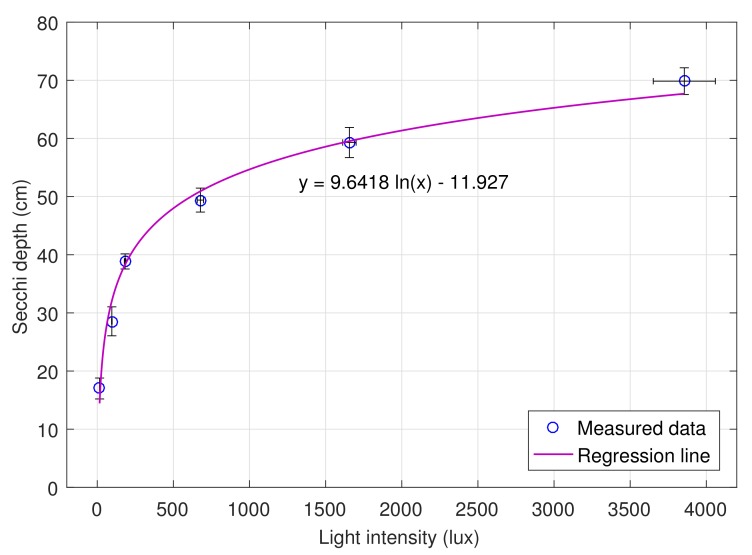
Regression line showing the relationship between Secchi depth and light intensity.

Based on these data, the regression function $y=9.6418\ln\left(x\right)-11.927$, as shown in Figure 9, was generated by using the trendline tool in Microsoft Excel with the coefficient of determination $R^{2}$ = 0.9864. Since $R^{2}$ is close to 1, the adequacy of the regression model is accepted in this case. Thus, measurements by using our instrument can replace manual measurements by using Secchi disk.

### 4.3. Results of ZSD Measured by Secchi Disk and by Instrument in Different Locations

Table 2 and Table 3 present the ZSDs obtained from two measurement methods, i.e., using Secchi disk and our instrument. Measurement experiments were implemented in Ho Chi Minh City and five provinces of Mekong Delta, Vietnam. Three observation locations, namely a reservoir, a shrimp pond, and a wastewater pond, were selected. We made ten measuring attempts in each location, and then calculated the average value.

We can see that, in the case of reservoir, the ZSDs of two measurement methods are very similar. However, their central tendencies are somewhat different. The mean percentage difference between the results measured by using Secchi disk and the results measured by using our instrument is 1.91% for reservoir, 1.94% for shrimp pond, and 2.64% for wastewater pond. However, for the reservoir, the maximum difference in six sites reaches 2.1 cm, corresponding to 4.88% and the minimum is 0.25 cm, corresponding to 0.53%. On the other hand, for the shrimp pond, the overall maximum difference tends to decrease, leading to an average of 1.4 cm, corresponding to an average of 1.56%. Lastly, for the wastewater pond, the highest overall difference is 0.6 cm (2.3%).

Therefore, the use of this instrument is expected to improve measurement precision with acceptable differences. The consistency between two measurement methods increases when the water environment gets worse due to the presence of suspended particles. It is noteworthy that even the most significant average difference is only 2.9%, indicating that the measurement precision is good. We should note that there is a small gap between the average percentage difference calculated from the results in Table 2 and Table 3 and that obtained from the results in Figure 9. Specifically, from the regression function, Δ= 2.56, 3.51, 0.55, 1.52, 0.24, 2.16; $\Delta_{p}=$ 17.73, 10.95, 1.44, 2.97, 0.4, 3.19; mean of $\Delta_{p}=$ 6.1%. In Table 2 and Table 3, Δ= 0.25, 0.72, 0.8, 1.4, 0.64, 0.87, 2.1, 0.72, 0.6, 0.9, 1.2, 0.7, 0.25, 0.72, 0.8, 0.7, 0.6, 0.3; $\Delta_{p}=$ 0.53, 1.90, 2.90, 2.69, 1.56, 3.48, 4.88, 2.05, 2.30, 1.53, 2.79, 3.18, 0.53, 1.90, 2.90, 1.27, 1.46, 1.07; mean of $\Delta_{p}=$ 2.16%. The main reason of this gap is that we could not collect huge number of samples in six sites (shrimp farms) because the owners of these farms were afraid that long-duration measurements could influence their production. In the future, we will evaluate the performance of our instrument with higher number of samples.

**Remark** **1.** *From the above reported results, we can see that the residuals of the regression function $y=9.6418\ln\left(x\right)-11.927$ are equal to 2.56, -3.51, 0.55, -1.52, -0.24, and 2.16. Predictions in a different location seem to outperform the regression function (reported differences are 0.25, 0.72, 0.8, 1.4, 0.64, 0.87, 2.1, 0.72, 0.6, 0.9, 1.2, 0.7, 0.25, 0.72, 0.8, 0.7, 0.6, and 0.3), even though one would expect the opposite to happen. This behavior may be attributed to the limited testing of the instrument due to technical reasons. Therefore, more experiments are needed to provide a clear picture on the reliability of the instrument.*

## 5. Conclusions

Water environment pollution is worsening; therefore, the requirement for continually monitoring water quality is in great need. This study aimed to develop an instrument using solar energy to support the automatic measurement of water clarity in place of manual measurement by Secchi disk. The instrument uses solar energy and can work autonomously. Besides, another advantage of this instrument is the ability to connect with other devices in IoT applications. Specifically, the measured light intensity is collected by a light sensor and processed directly by a controller. Then, the data of Secchi depth are sent and stored in a cloud server so that users can read these data through a mobile application for better management decisions. Experiment results show that our instrument based measurements are tightly consistent with the manual measurements using the Secchi disk. Moreover, using our instrument gives measurement results more quickly than using conventional Secchi disk because it is not affected by environmental conditions and does not require experienced workers to operate at all times. Therefore, this instrument can efficiently replace the Secchi disk in water clarity measurement. The manufacturing cost of our instrument is approximate USD 500. It can withstand wave heights less than 35 cm in aquaculture ponds and lakes. Since the structure of our proposed instrument is simple, maintenance service mainly deals with maintaining mechanical components one per three months. Our instrument is expected to operate normally for at least five years. The weakest components of this instrument are those submerged in salt water. Although being made of stainless steel, the quality of these components may be degraded by time.

As future works, we will extend this instrument to deeper depths by using a longer lead screw and equip it with multiple sensors to monitor a broader range of water quality parameters such as pH, temperature, dissolved oxygen, etc.

## Figures and Tables

**Table 1:** Secchi depth (ZSD, cm) measured with naked eye and light intensity (LI, lux) measured with our instrument for generating regression function.

Experiment	First Experiment		Second Experiment		Third Experiment		Fourth Experiment		Fifth Experiment		Sixth Experiment	
**No.**	**ZSD**	**LI**	**ZSD**	**LI**	**ZSD**	**LI**	**ZSD**	**LI**	**ZSD**	**LI**	**ZSD**	**LI**
**1**	15.5	14	28.5	94	38	192	50.5	660	57.5	1615	67.5	4149
**2**	16.5	16	27.5	100	37.5	189	47.5	661	59.5	1716	69	3987
**3**	14.5	15	24.5	96	38.5	183	52	643	58.5	1635	72	3532
**4**	15	14	31.5	96	39	190	48	652	60.5	1598	68	4015
**5**	20	16	28.5	97	39.5	181	50	678	63	1637	67	3884
**6**	16.5	15	28.5	95	41	179	52	704	57.5	1662	69.5	3647
**7**	18	16	27	93	40	177	46.5	691	64.5	1713	72	3582
**8**	20	17	32.5	96	37.5	182	48.5	688	57.5	1677	73.5	3749
**9**	17.5	15	31.5	94	40.5	180	52	701	55.5	1599	67.5	4036
**10**	16.5	16	25.5	97	37	177	47	695	59	1710	72.5	3937
**Mean**	17	15.4	28.55	95.8	38.85	183.0	49.4	677.3	59.3	1656.2	69.85	3855.4
**Standard** **deviation**	1.8	0.92	2.49	1.9	1.3	5.18	2.06	20.7	2.59	44.0	2.3	204
**CV (%)**	10.59	5.95	8.72	2.0	3.35	2.83	4.17	3.05	4.37	2.7	3.29	5.3

**Table 2:** Secchi depth measured by the Secchi disk and our instrument in HCM City, Soc Trang, and Bac Lieu provinces.

Location	Can Gio District, Ho Chi Minh City			Vinh Chau District, Soc Trang Province			Gia Rai District, Bac Lieu Province		
**Measurement**	**ZSD, cm**			**ZSD, cm**			**ZSD, cm**		
	**Reservoir**	**Shrimp** **Pond**	**Wastewater ** **Pond**	**Reservoir**	**Shrimp** **Pond**	**Wastewater** **Pond**	**Reservoir**	**Shrimp** **Pond**	**Wastewater** **Pond**
**Using the** **Secchi disk**	47	38	28	52	41	25	43	35	26
**Using the** **instrument**	46.75	37.28	27.2	53.4	40.36	24.13	45.1	35.72	25.4
**Absolute** **difference**	0.25	0.72	0.8	1.4	0.64	0.87	2.1	0.72	0.6
**Percentage** **difference**	0.53	1.90	2.90	2.69	1.56	3.48	4.88	2.05	2.30

**Table 3:** Secchi depth measured by Secchi disk and our instrument in Ben Tre, Tra Vinh, and Ca Mau provinces.

Location	Thanh Phu District, Ben Tre Province			Duyen Hai District, Tra Vinh Province			Cai Nuoc District, Ca Mau Province		
**Measurement**	**ZSD, cm**			**ZSD, cm**			**ZSD, cm**		
	**Reservoir**	**Shrimp** **Pond**	**Wastewater ** **Pond**	**Reservoir**	**Shrimp** **Pond**	**Wastewater** **Pond**	**Reservoir**	**Shrimp** **Pond**	**Wastewater** **Pond**
**Using the** **Secchi disk**	59	43	22	40	33	19	55	41	28
**Using the** **instrument**	58.1	41.8	21.3	39.75	37.28	18.2	54.3	40.4	27.7
**Absolute** ** difference**	0.9	1.2	0.7	0.25	0.72	0.8	0.7	0.6	0.3
**Percentage** ** difference**	1.53	2.79	3.18	0.53	1.90	2.90	1.27	1.46	1.07
